# Supplementary material for: Urban segregation on multilayered transport networks: a random walk approach
Source: Sci Rep. 2024 Apr 10;14:8370. doi: 10.1038/s41598-024-58932-9 (PMC11006669; doi:10.1038/s41598-024-58932-9)
Supplement: Supplementary file 1 — Supplementary Figures. [file 41598_2024_58932_MOESM1_ESM.pdf]

Supplementary Material

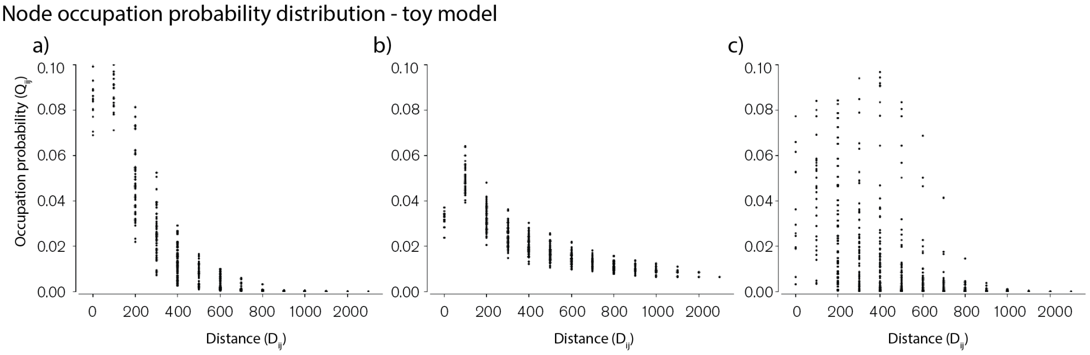

Figure 1: Plot of Occupation Probability (Q_{ij}) vs. Distance (D_{ij}) in a Network: This graph illustrates how the probability that a random walk starting from node i will pass through node j (Q_{ij}) varies with the distance (D_{ij}) between the two nodes in the network. Each point on the plot represents a pair of nodes, showing that the likelihood of node j being visited in a walk originating from node i generally decreases as the nodes become further apart. Each graph represents a different random walk type, where a) represents the normal random walk case, b) the preferential random walk where the probability of transitioning to node j is proportional to it's betweenness centrality, and c) the Lévy flight case.

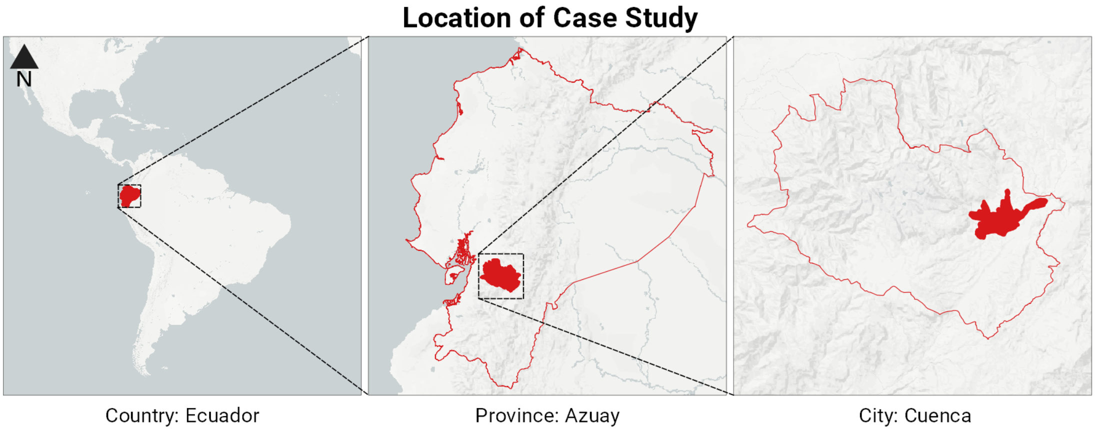

Figure 2: Location the case study: Cuenca, Ecuador

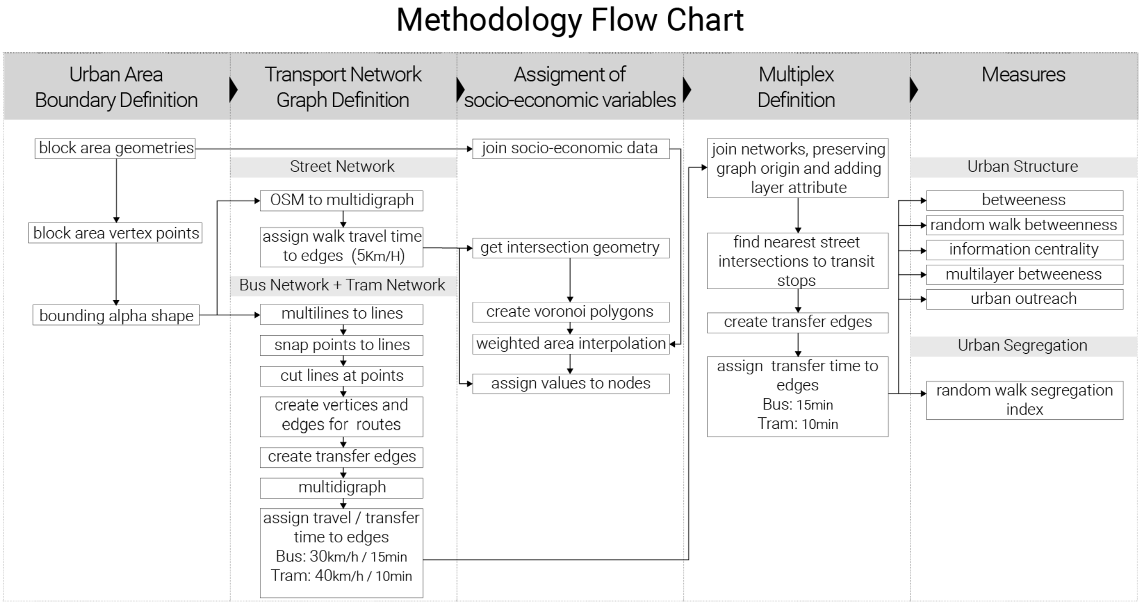

Figure 3: Flow chart of methodology employed in the case study of Cuenca, Ecuador. Steps include 1) creating a clear spatial boundary for city. 2) preprocessing all input data regarding the different transport networks to create the multilayer definition for the city. 3) assigning socio-economic variables to the street nodes using voronoi tessellation and area-weighted spatial joins. 4) measuring structural properties of the multilayer network. and 5) calculating the random walk segregation index as proposed in th study.

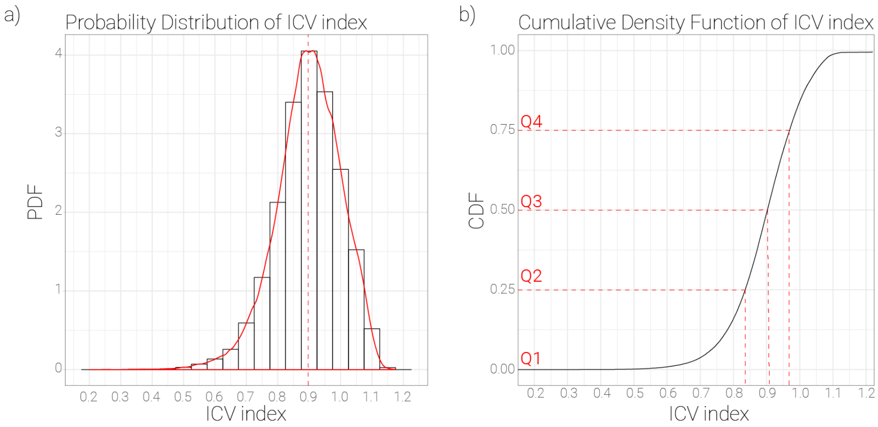

Figure 4: Statistical distribution of ICV index values in Cuenca. The index of life conditions (ICV) ranges from $0-2$ where households with less the 1 express deprivation and above one present life conditions above standard. Most individuals have ICV values less than one, showing that they do not meet the minimum threshold for well-being across one or more variable. The ICV values across the population follows a normal distribution with a mean of 0.9.

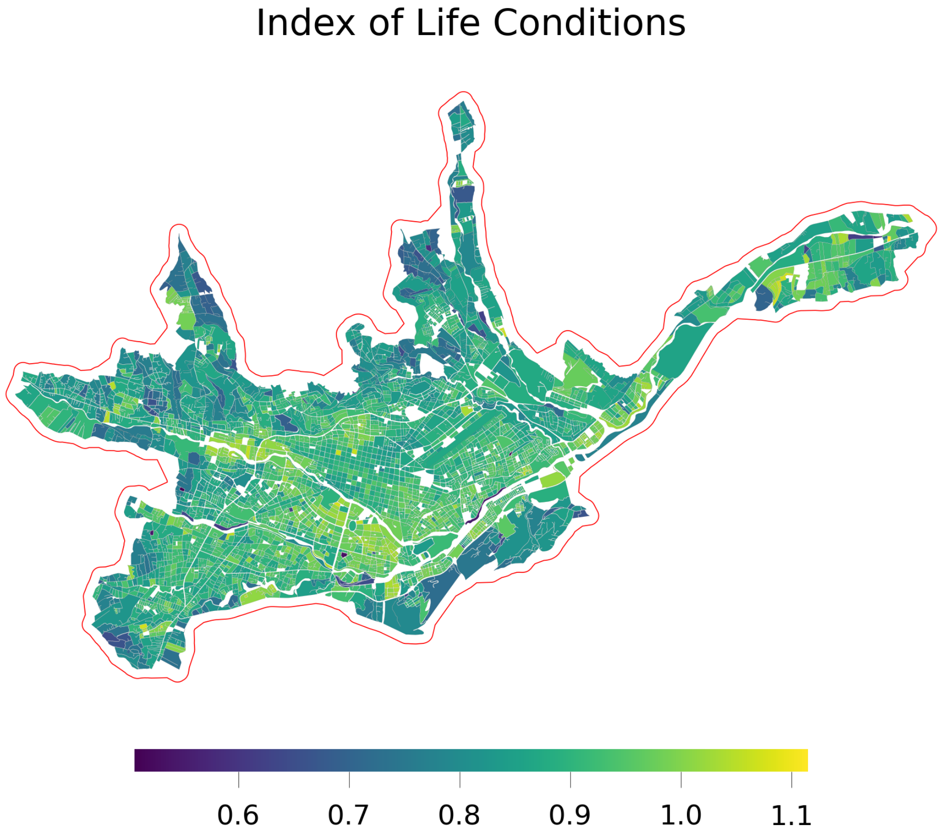

Figure 5: Spatial distribution of index of life conditions in Cuenca, Ecuador. ICV values are aggregated at the urban block level and the median is taken as the representative value for the block. Higher ICV values are present along the river margins following the east-west axis of the city.

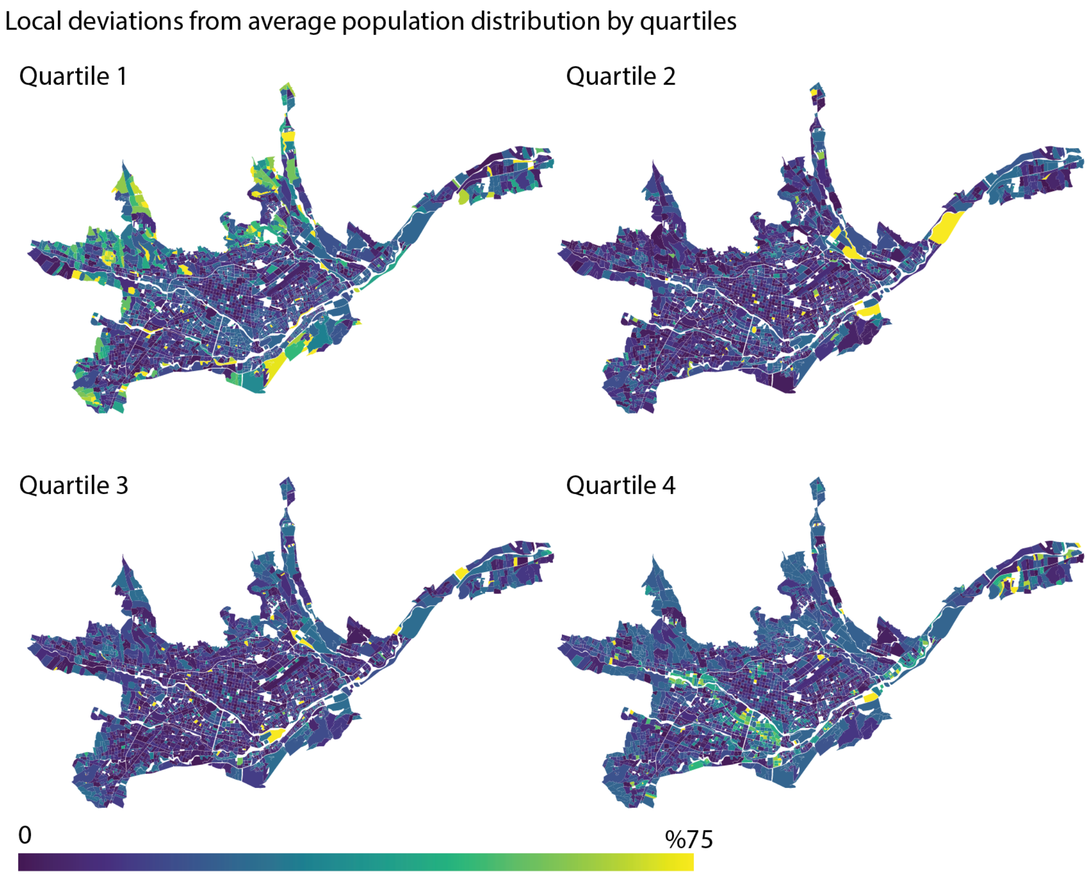

Figure 6: This series of maps visualises the local deviations from the city's average population distribution, segmented by quartiles. By calculating the difference between the city-wide population distribution and that of individual blocks, we identify areas of significant demographic divergence. Notably, the maps reveal a pronounced over-representation of lower quartile groups on the city's periphery. Conversely, upper quartile groups show a marked over-representation along the riverbanks. This spatial analysis underscores the demographic disparities within the city, highlighting how socio-economic factors might be influencing residential patterns.

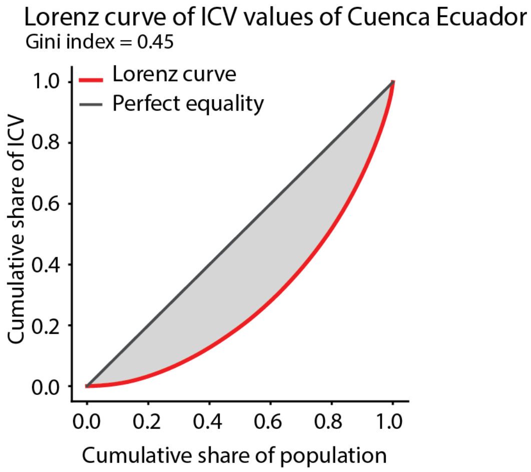

Figure 7: Lorenz Curve of index of life conditions (ICV) Values with a Gini Index of 0.45. This graph illustrates the distribution of ICV values across the city of Cuenca, highlighting the inequality in variation levels.

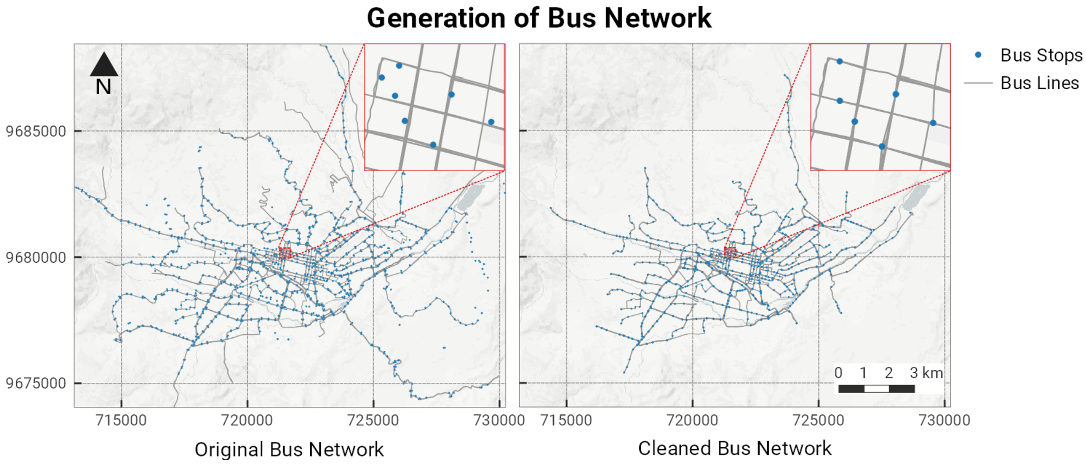

Figure 8: Left: Original shape files of bus lines and bus stops in the city. Right: Final Bus Network after cleaning geometries and creating network. To create the networks representing both the bus and tram the line geometries were processed to ensure all routes were digitised as one single continuous line. The point geometries did not necessarily lie on the line geometries, and no field contained which line a particular stop belonged to. To solve this, for each route a buffer distance of $50m$ was set and all points within this buffer where snapped to that particular route. Once the geometries of the routes and stops/stations were matched, a function was developed to create a multidigraph of each route. This process was done by cutting the line geometries by the points and creating the corresponding vertices and edges. Vertex ids were set to reflect which network they belong to. Geometric properties were conserved for visualisation purposes, and a temporal weight was added to each edge by calculating travel time using distance and a average travel speed of $30Km/h$ for the bus network and $40Km/h$ for the tram network. After creating a multidigraph for each route for the bus and tram network, transfer edges were created for routes within each that shared the same stop/station. These transfer edges were weighted by an average waiting time of 7 minutes for bus network and 5 minutes for the tram network. The resulting graphs are: a strongly connected multidigraph for the bus network, defined as $G_b = (N_b, L_b, w_b)$; and a strongly connected multidigraph for the tram network, defined as $G_t = (N_t, L_t, w_t)$, and described through their corresponding adjacency and time-weighted adjacency matrix.

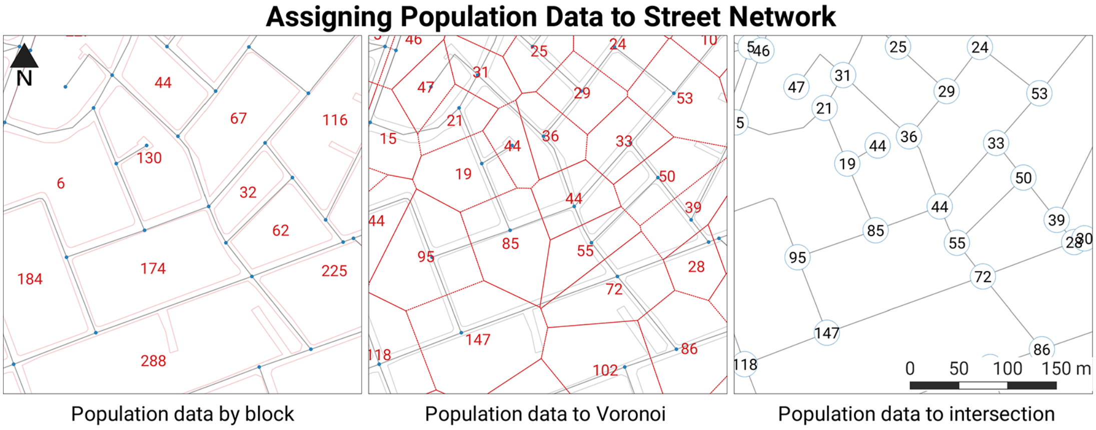

Figure 9: Assigning socio-economic data from blocks to street intersections. In order to measure urban segregation within the multilayer network, socio-economic data needs to be incorporated into the street network vertices. The most complete dataset for socio-economic variables is census data, and the finest spatial resolution that can be achieved is at block-level. In order to assign the block level data to the street intersection the amount of population that blocks contribute to each intersection needs to be calculated. First a Voronoi tessellation is created using the street intersection geometries, then a weighted area overlay interpolation is used to calculate population values for the Voronoi polygons. Finally, Voronoi polygon values are assigned to their corresponding vertex in the street network. For simplicity, this assumes that each individual living in a particular block will always start a journey through the city from the same intersection.

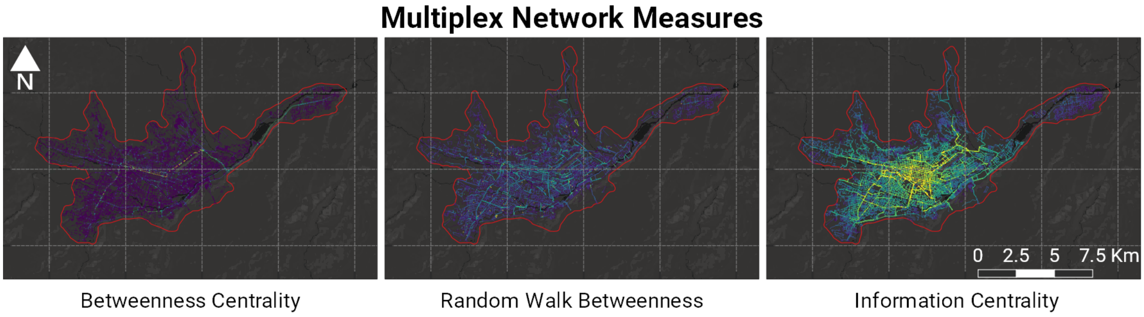

Figure 10: Spatial distribution of multilayer centrality measures.

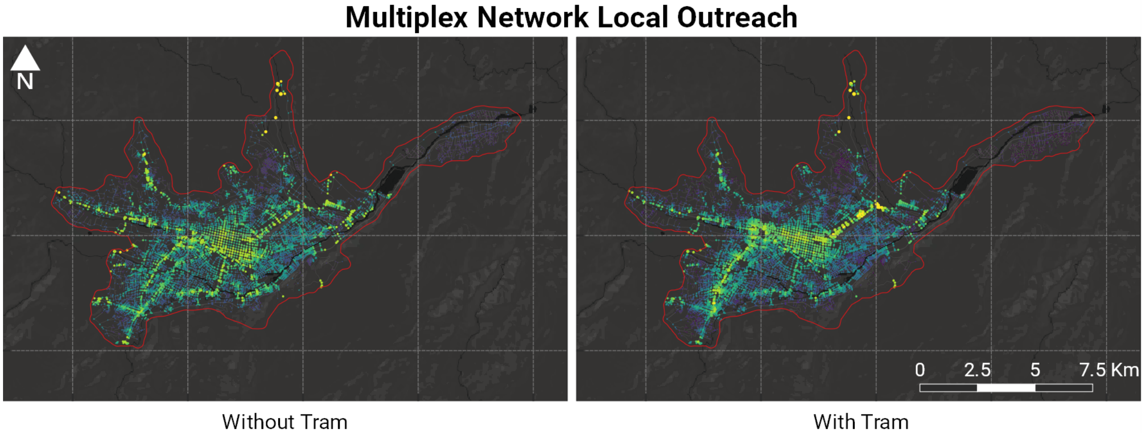

Figure 11: For M_{sbt} the local outreach of a vertex $i \in V_s$ is defined as: $L_\tau(i) = \frac{1}{N(\tau)} \sum_{j|\tau_m(i,j)<\tau} d(i,j)$ where $d(i,j)$ is the distance between vertex i and j, and $N(\tau)$ is the number of nodes reachable on the multilayer network within a given travel cost τ. Through this measure we quantify the accessibility of every $i \in V_s$ given for a specific temporal constraint, and can calculate how the introduction of the tram network changes the accessibility of i. Although this measure does not influence the calculation of the segregation index, it helps in identifying how the addition of specific layers to the multilayer network change local accesbility.

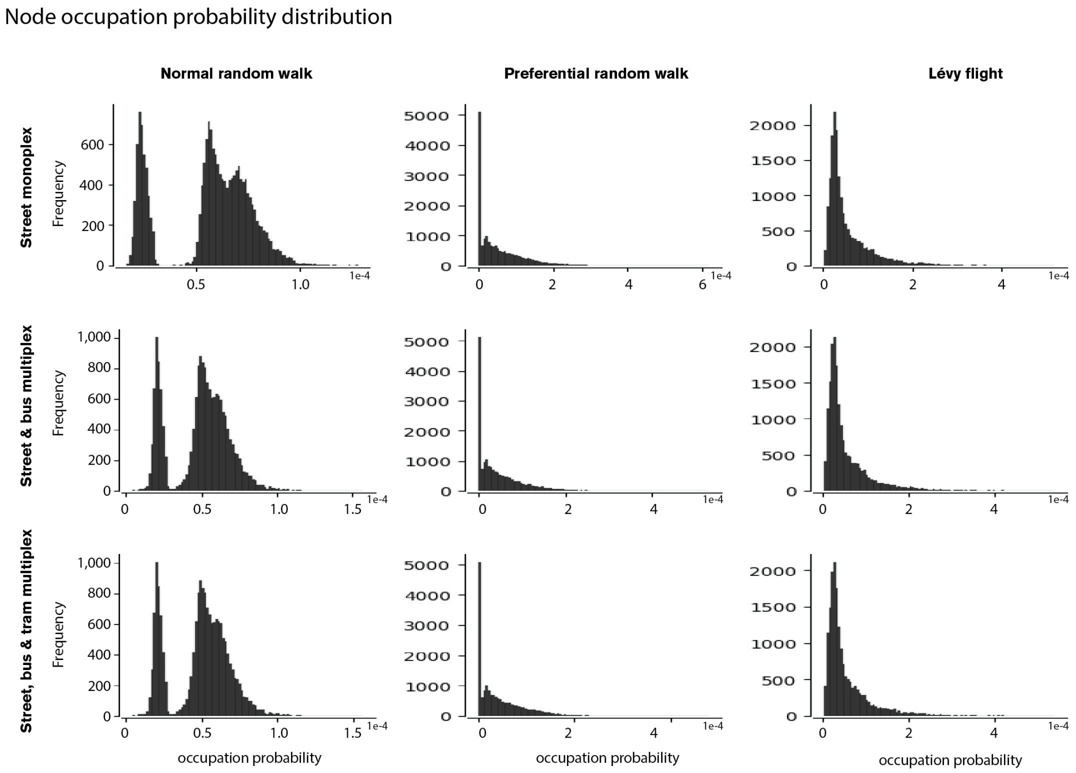

Figure 12: Histograms of Node Occupation Probability Distributions Across Random Walk Types and Network Configurations for the case study of Cuenca, Ecuador: This graph presents a comparative analysis of node occupation probabilities for normal, preferential, and Lévy flight random walks, segmented by the different types of the network considered. From the simplest street-only network to the more integrated networks that include bus and tram lines, the histograms reveal how the structure of the network influences the spread and concentration of node occupation probabilities. Notably, the Lévy flight random walks exhibit a power-law distribution across all network types, indicating a characteristic long-tail where a small number of nodes are highly frequented, while most others are less so. This pattern highlights the Lévy flight's propensity for exploring both local neighbourhoods and distant nodes, contrasting with the more uniform or centrality-biased distributions seen in normal and preferential walks, respectively.
